# The Plasma Membrane-Localized Sucrose Transporter IbSWEET10 Contributes to the Resistance of Sweet Potato to *Fusarium oxysporum*

**DOI:** 10.3389/fpls.2017.00197

**Published:** 2017-02-14

**Authors:** Yan Li, Yannan Wang, Huan Zhang, Qian Zhang, Hong Zhai, Qingchang Liu, Shaozhen He

**Affiliations:** Beijing Key Laboratory of Crop Genetic Improvement/Laboratory of Crop Heterosis and Utilization, Ministry of Education, China Agricultural UniversityBeijing, China

**Keywords:** *IbSWEET10*, plasma membrane, sucrose transporter, *Fusarium oxysporum*, sweet potato

## Abstract

SWEET (Sugars Will Eventually be Exported Transporter) proteins, a novel family of sugar transporters, mediate the diffusion of sugars across cell membranes and acts as key players in sucrose phloem loading. Manipulation of SWEET genes in plants leads to various effects on resistance to biotic and abiotic stresses due to disruption of sugar efflux and changes in sugar distribution. In this study, a member of the SWEET gene family, *IbSWEET10*, was cloned from the sweet potato line ND98. mRNA expression analysis in sweet potato and promoter β-Glucuronidase analysis in *Arabidopsis* showed that *IbSWEET10* is highly expressed in leaves, especially in vascular tissue. Transient expression in tobacco epidermal cells revealed plasma membrane localization of IbSWEET10, and heterologous expression assays in yeast indicated that *IbSWEET10* encodes a sucrose transporter. The expression level of *IbSWEET10* was significantly up-regulated in sweet potato infected with *Fusarium oxysporum* Schlecht. f. sp. *batatas.* Further characterization revealed *IbSWEET10*-overexpressing sweet potato lines to be more resistant to *F. oxysporum*, exhibiting better growth after infection compared with the control; conversely, RNA interference (RNAi) lines showed the opposite results. Additionally, the sugar content of *IbSWEET10*-overexpression sweet potato was significantly reduced, whereas that in RNAi plants was significantly increased compared with the control. Therefore, we suggest that the reduction in sugar content caused by *IbSWEET10* overexpression is the major reason for the enhanced *F. oxysporum* resistance of the transgenic plants. This is the first report that the IbSWEET10 transporter contributes to the resistance of sweet potato to *F. oxysporum*. The *IbSWEET10* gene has the great potential to be used for improving the resistance to *F. oxysporum* in sweet potato and other plants.

## Introduction

Sugars are essential substrates for the fundamental processes of plant growth, the major energy sources for generating adenosine triphosphate (ATP), and the main precursor for various storage carbohydrates (Eveland and Jackson, 2012; Guo et al., 2014). In addition, sugars have important roles in signal transduction in higher plants and can regulate many developmental and physiological processes by influencing expression of different sets of genes in various pathways (Koch, 1996; Gibson, 2000; Rolland et al., 2006). The responses of plants to sugar levels also help to integrate metabolism, growth, development and environmental stress responses (Huang et al., 2014). In plants, carbohydrates are synthesized in source leaves, mainly in the form of sucrose and are translocated to sink tissues to sustain heterotrophic metabolism and growth (Roitsch and Gonzalez, 2004). Indeed, sucrose metabolism, partitioning and sensing are proven to be pivotal for many plant processes such as flowering (Roldán et al., 1999), seed germination (Finkelstein and Lynch, 2000), tuber formation (Mullerrober et al., 1992), nutrient synthesis (Forde, 2002) and responses to biotic stresses (Lemoine et al., 2013).

Long-distance transport of sugar to sink organs occurs via the vascular system, which begins with sucrose loading through the sieve element-companion cell complex (SE/CC) by plant sugar transporters, such as SUTs /SUCs (disaccharide transporters, DSTs) and MSTs (monosaccharide transporters). Functioning as sugar/proton symporters, both DSTs and MSTs belong to the major facilitator superfamily (MFS), the members of which possess 12 transmembrane (TM) helices (Sauer et al., 1990; Marger and Saier, 1993; Sauer and Stolz, 1994; Boorer et al., 1996; Zhou et al., 1997). At least nine DSTs and 53 MSTs are encoded by the *Arabidopsis* genome, with most being localized to the plasma membrane (Williams et al., 2000; Lalonde et al., 2004; Büttner, 2007). AtSUC2 and -4 are involved in sucrose phloem loading in source leaves; AtSUC2 is mainly expressed in collection and transport phloem and AtSUC4 in minor veins (Weise et al., 2000; Srivastava et al., 2008). Tissue-specific expression of AtSUCs plays key roles in the sugar partitioning of different organs, eventually affecting their development, some examples are AtSUC1 in pollen (Sivitz et al., 2008), AtSUC5 in endosperm (Baud et al., 2005) and AtSUC9 in flowers (Sivitz et al., 2007). In addition to roles in plant development, SUTs respond to abiotic stresses and therefore contribute to the environmental tolerance of plants. The transcript levels of certain genes, such as *AgSUT1* in celery (Noiraud et al., 2000) and *OsSUT2* in rice (Ibraheem et al., 2011), are significantly altered in response to salt or drought stress. Furthermore, RNA interference (RNAi) of *PtaSUT4* in *Populus tremula* resulted in delayed wilting during acute drought stress (Frost et al., 2012). Although the role of the SUT family in plant abiotic tolerance is generally attributed to their functions in sugar transport and compartmentalization, further investigation of the detailed mechanisms is needed.

The SWEET (Sugars Will Eventually be Exported Transporter) family comprises a newly described family of sugar transporters that are ubiquitous in prokaryotes, plants and animals. As uniporters, SWEET proteins mediate uptake or efflux of various mono- and disaccharides across the plasma membrane or the tonoplast and exhibit low sugar affinity (Chen et al., 2010). Usually, SWEET proteins are classified into three major types based on the number of 3-TM domains, semiSWEET (bacteria) with a single 3-TM domain, SWEET (eucaryotes) with a linker in the middle of two 3-TM domains (7-TM) and extraSWEET (Vv14G09070) with the duplication of 7-TM (Xuan et al., 2013; Patil et al., 2015). In addition, Hu et al. (2016) identified a fusion of archaeal and bacterial SemiSWEETs to form eukaryotic SWEETs in *Batrachochytrium dendrobatidis*. Members of the SWEET family underwent expansion during plant evolution. For example, the genomes of *Chlamydomonas reinhardtii* and *Physcomitrella patens* harbor 5 and 6 SWEET genes, respectively, whereas 8 are present in *Amborella trichopoda*, the earliest divergent angiosperm, 47 and 52 are found in the dicotyledon *Eucalyptus grandis* and soybean (Eom et al., 2015; Patil et al., 2015). The *Arabidopsis* genome contains 17 members of the SWEET family, which can be grouped into four phylogenetic subclades, and 21 members have been identified in rice (Chen et al., 2010).

Variation among SWEETs in different subclades in *Arabidopsis* may indicate their sugar transport preferences. Clade I SWEETs mainly transport 2-deoxyglucose, whereas clade II prefers glucose. All members in clade III utilize sucrose as a substrate, and AtSWEET16 and -17 in clade IV act as fructose uniporters (Chen L.Q. et al., 2015). The functions of AtSWEETs have been studied extensively. AtSWEET1, -5, -8, and -9 are involved in the development of reproductive organs such as pollen (tube) and nectar (Engel et al., 2005; Guan et al., 2008; Chen et al., 2010; Lin et al., 2014). AtSWEET4, -11, -12, -16, and -17, have essential roles in the growth of aerial parts or roots as well as abiotic resistance by influencing sugar partitioning (Chen et al., 2012; Klemens et al., 2013; Guo et al., 2014; Liu et al., 2016). Overexpression of *OsSWEET5* led to growth retardation and precocious senescence of seedlings by regulating auxin signaling pathway in rice (Zhou et al., 2014). ZmSWEET4c and OsSWEET4 are key players for hexose transport across the basal endosperm transfer layer during seed filling in maize and rice, respectively (Sosso et al., 2015). Kanno et al. (2016) found that AtSWEET13 and AtSWEET14 mediated cellular gibberellin (GA) uptake, suggesting they might modulate GA responses in *Arabidopsis*. The *PsSWEET* genes were actively expressed during seed development and germination in pea (Jameson et al., 2016). The interactions of *SWEET* and *CWINV* genes and cytokinins led to the loss of apical dominance and the appearance of multiple shoots after infection by *Rhodococcus fascians* in pea (Dhandapani et al., 2016). *OsSWEET11* (*Os8N3*) and *OsSWEET14* (*Os11N3*) which were characterized to be the susceptibility (S) genes mediated the resistance to *Xanthomonas oryzae* pv. *Oryzae* in rice (Yang et al., 2006; Liu et al., 2011; Yuan and Wang, 2013). *AtSWEET2* encodes a vacuolar glucose transporter that prevents sugar loss from roots and thus contributes to resistance to *Pythium irregulare* (Chen H.Y. et al., 2015). These findings indicate that the SWEET genes have multiple functions and play important roles in the processes of plant growth and development and resistance to biotic/abiotic stresses.

Sweet potato [*Ipomoea batatas* (L.) Lam.] is the seventh most important staple crop (Zhai et al., 2016) and one of the most important root crops in the world, grown on at least 8 million hectares in 114 countries worldwide (Khoury et al., 2015; Zhang et al., 2016); however, its yield is severely limited by pathogens (Feng et al., 2011). As biotic pathogens obtain nutrients from their hosts for growth and reproduction and cause diseases, the nutritional relationship between pathogens and hosts is an important aspect of infection (Hancock and Huisman, 1981). *Fusarium*, a soil-borne fungus, which infects plant roots through wounds (Keane, 2012), typically acquires nutrients from the host for spore germination, and carbon is a major nutrient in this process (Baker, 1968). One species, *Fusarium oxysporum* Schlecht. f. sp. *batatas* causes fusarium wilt or stem rot of sweet potato, a major disease that limits the production of this crop (Clark et al., 1998). It has become one of the most serious diseases threatening sweet potato production in southern China. This pathogen can persist in soil for many years, invading plants through the seedling base, wounded roots or seeds, resulting in wilt disease or even death (Clark, 1994). Developing disease-resistant varieties is an important objective of sweet potato breeding. However, little work has been performed on the cloning of *F. oxysporum* resistance-associated genes in sweet potato and the mechanism of disease resistance is not clear. In sweet potato, the SWEET genes have not been reported and their functions remain to be elucidated.

In this study, we cloned a member of the SWEET family from sweet potato, *IbSWEET10*, a gene that encodes a sugar transporter protein involved in sucrose transport. β-Glucuronidase (GUS) analysis revealed its leaf-dominant expression, particularly in vascular tissue in *Arabidopsis*. IbSWEET10-GFP analysis indicated plasma membrane localization of the IbSWEET10 protein in tobacco and yeast, respectively. Moreover, we developed *IbSWEET10*-overexpressing and RNA-interference sweet potato lines and analyzed its contribution to the resistance to *F. oxysporum* in sweet potato. The findings will contribute to the improvement of disease resistance in sweet potato through molecular breeding.

## Materials and Methods

### Plant Materials

Sweet potato line ND98 and cv. Lizixiang were cultivated in a greenhouse under a regime of 16 h light and 8 h darkness (28°C). ND98 is resistant to *F. oxysporum* and Lizixiang is susceptible to this disease. ND98 was used for gene cloning in this study. One expressed sequence tag (EST) selected from the ND98 EST library constructed at our laboratory was up-regulated after the infection of *F. oxysporum* in ND98 and was used for gene cloning. The cloned gene was further introduced into Lizixiang for function characterization. *Arabidopsis thaliana* plants (Col-0) were grown in a 16-h light/8-h dark climate-controlled chamber at 22°C for analyzing the promoter of this gene.

### Cloning and Sequence Analysis of *IbSWEET10* and the 5′-Promoter Region

Total RNA was extracted from the fresh leaves of 4-week-old *in vitro*-grown plants of ND98 using RNAprep Pure Plant Kit (Tiangen Biotech, Beijing, China). The RNA samples were reverse-transcribed according to the instructions of PrimeScript^TM^ II 1st Strand cDNA Synthesis Kit (TaKaRa, Beijing, China). Rapid Amplification of cDNA Ends (RACE) was then applied to amplify the full-length sequence of *IbSWEET10* using SMARTer RACE 5′/3′ Kit (TaKaRa, Beijing, China). Gene-specific primers (GSPs) were used for 5′ RACE (5GSP1 and -2) and 3′ RACE (3GSP1 and -2). Amplification of the genomic sequence of *IbSWEET10* with GS-F/R primers was performed using genomic DNA extracted from the fresh leaves of 4-week-old *in vitro*-grown ND98 plants, and the 5′-promoter region of *IbSWEET10* was isolated using the GSPs (GW1 and -2) and Genome Walking Kit (TaKaRa, Beijing, China). All polymerase chain reaction (PCR) products were subcloned into the pMD19-T vector (TaKaRa, Beijing, China) and then transformed into competent *Escherichia coli* strain DH5α cells and sequenced. The primers used in this study are listed in Supplementary Table S1.

The open reading frame (ORF) of *IbSWEET10* was predicted using ORF Finder^1^. The molecular weight and isoelectric point (*p*I) of the IbSWEET10 protein were calculated at http://web.expasy.org/compute_pi/. Multiple sequence alignment of the IbSWEET10 protein with other SWEET10 proteins was performed using DNAMAN software. MEGA 6.0 software was used to construct the phylogenetic tree with the neighbour-joining (NJ) method.

### Expression Analysis of *IbSWEET10* in Sweet Potato and *Arabidopsis*

Total RNA was isolated from roots, stems, leaves and petioles of 4-week-old *in vitro*-grown ND98 plants and storage roots, fibrous roots, stems, leaves and petioles of 3-month-old ND98 plants in the field. Total RNA was extracted as described above, and first-strand cDNA was synthesized using the PrimeScript^TM^ RT Reagent Kit with gDNA Eraser (Perfect Real Time; TaKaRa, Beijing, China). Quantitative real-time PCR (qRT-PCR) was performed to determine the transcript levels of *IbSWEET10* using SYBR Premix Ex Taq (Tli RNaseH Plus; TaKaRa, Beijing, China) and the 7500 Real-Time PCR system (Applied Biosystems, Foster City, CA, USA). The primers used to amplify *IbSWEET10* and *IbActin* (internal control, AY905538) are listed in Supplementary Table S1.

To generate the P*_Ibsweet10_*-GUS construct, the *IbSWEET10* promoter fragment containing the 5′ untranslated region (5′UTR) was amplified from the ND98 genomic DNA and subcloned into the expression vector pMDC162 at *Pac*I and *Asc*I sites. The reconstructed plasmid was introduced into the GV3101 strain of *A. tumefaciens*. *Arabidopsis* plants were transformed using the floral dip method (Clough and Bent, 1998), and transformants were identified on 1/2 MS medium containing 60 mg L^-1^ hygromycin.

The response of *IbSWEET10* to *Fusarium* infection was investigated in ND98 plants. *F. oxysporum* was inoculated onto solid potato dextrose agar (PDA) medium at 28°C and cultured for 1 week in the dark. When the mycelium was fully expanded on the plate, the medium was minced, and the agar plugs were transferred to sterile distilled water and shaken for 2 h to loosen the spores from the mycelium surface. The resulting spore suspension was filtered through a nylon mesh to remove solid material; the spore concentration was determined using a haemocytometer and adjusted to ∼10^7^ spores per mL. Before being grown in 19-cm-diameter pots containing sterile sandy loam, 20-cm-cuttings of ND98 with an apical bud from the field were soaked in spore suspension for 30 min. Each pot contained three cuttings as one treatment. All leaves of each treatment were collected at 0, 3, 5, 7, and 9 days post-inoculation (dpi), and expression of *IbSWEET10* was assessed by qRT-PCR.

### Subcellular Localization of IbSWEET10

The ORF of *IbSWEET10* (stop codon deleted) was amplified using primers 83S-F/R and subcloned into a modified pMDC83 vector at *Spe*I and *Asc*I restriction sites. The fusion construct (*IbSWEET10-GFP*) and the empty vector (*GFP*) were separately transformed into the EHA105 strain of *Agrobacterium tumefaciens*, and each transformant was co-infiltrated with a plasma membrane-localized marker (CD3-1007; Nelson et al., 2007) into the leaves of *Nicotiana benthamiana*, as previously described by Strasser et al. (2007). Furthermore, the sequence of *IbSWEET10* with *GFP* was amplified from *IbSWEET10-GFP* fusion construct using primers SG-F/R and subcloned into the pDR196 vector at *Sma*I and *Xho*I restriction sites (Rentsch et al., 1995). The constructed vector was transformed into the sucrose transport deficient *Saccharomyces cerevisiae* mutant SUSY7/ura3 (Riesmeier et al., 1992) for subcellular localization assay. To select positive transformants, the transformed yeast cells were grown on yeast culture medium containing 6.7 g L^-1^ yeast nitrogen base, 2 g L^-1^ amino acid (uracil) drop out mix and 20 g L^-1^ glucose as the sole carbon source. The agroinfiltrated tobacco leaves and the yeast transformants were visualized with a laser scanning confocal microscope (Nikon Inc., Melville, NY, USA).

### Functional Analysis of *IbSWEET10* in Yeast

The ORF of *IbSWEET10* was amplified using primers 196-F/R and then subcloned into the yeast expression vector pDR196 at *Sma*I and *Xho*I restriction sites (Rentsch et al., 1995). As a positive control, the *AtSUT4* gene was also cloned into pDR196 (Weise et al., 2000). The constructed vectors and the empty vector were transformed into the sucrose transport deficient *S. cerevisiae* mutant SUSY7/ura3 (Riesmeier et al., 1992) for complementation assays. The positive transformants were selected as described above and were then cultured on medium containing 20 g L^-1^ sucrose to assess the function of *IbSWEET10*. The average diameters of yeast colonies were measured after 1 week using the ImageJ software. In addition, the hexose transport-deficient *S. cerevisiae* mutant EBY.VW4000 (Wieczorke et al., 1999) and drop tests were used to examine whether IbSWEET10 transports hexose. The transformants were grown overnight to an optical density at 600 nm (OD_600_) of 0.6 in liquid medium containing 20 g L^-1^ maltose as the sole carbon source, and serial dilutions (OD_600_ = 0.6, 0.06, 0.006, 0.0006, and 0.00006) were plated on solid media containing different carbon sources (glucose, fructose, galactose, and mannose) for 1 week at 30°C.

### Production of Transgenic Plants

To explore the function of *IbSWEET10*, the expression cassette P_35S_-*IbSWEET10-*T_NOS_ was inserted into the plant binary vector pCAMBIA3301 at *Sac*I and *Xba*I sites to generate the overexpression plasmid pC*IbSWEET10*. To construct the RNAi plasmid pF*IbSWEET10*, two fragments (FF and RF) were amplified from the coding sequence of *IbSWEET10* using the primers Si-UF/R and Si-DF/R and inserted into the vector pFGC5941 at *Xho*I/*Swa*I and *Bam*HI/*Xba*I sites, respectively. The sequence-verified plasmids pC*IbSWEET10* and pF*IbSWEET10* and the empty vector were separately transfected into *A. tumefaciens* strain EHA105. The transformation, regeneration, and overexpression lines (OX) identification of sweet potato cv. Lizixiang plants were performed as described by Wang et al. (2016). To identify *IbSWEET10* RNAi lines, genomic DNA was extracted from the leaves of putative transgenic plants, and PCR was performed using the primers int-F/R. The transformants with the empty vector pCAMBIA3301 (VCo) and ones with the empty vector pFGC5941 (VCi) were used as the controls for the OX and RNAi lines, respectively.

### Histochemical Analysis of GUS Activity

β-Glucuronidase reporter gene activity in transgenic *Arabidopsis* was examined by histochemical staining as described by Guo et al. (2014). The incubation time for whole seedlings, roots, and flowers was 2 h; 6 h was used for leaves, stems, siliques, and seeds.

### *In vitro* Assay for Sucrose Tolerance

Transgenic and control sweet potato plants were cultured on solid MS medium with different sucrose concentrations (0, 1, 2, 3, 4, 5, and 6%) at 28±1°C under 16 h of cool-white fluorescent light at 54 μmol m^-2^ s^-1^. The standard sucrose concentration of MS medium is 3%. The growth status was observed after 4 weeks, and the fresh weight (FW) was measured immediately.

### Inoculation of Transgenic Sweet Potato with *F. oxysporum*

Transgenic and control sweet potato plants were transferred to the soil in a greenhouse and a field and their 20-cm-cuttings were inoculated with *F. oxysporum* using the same methods described above to evaluate the resistance to *F. oxysporum*. Growth of the transgenic and control lines was evaluated at 9 or 7 dpi, and stem samples were collected for histological examination according to the method of Zhang et al. (2014).

### Analysis of Sugar Content

To determine changes in sugar composition during *Fusarium* infection, leaves of transgenic and control plants were harvested at 0 and 9 dpi (or 7 dpi) for quantification of sucrose, glucose, and fructose using high-performance liquid chromatography (HPLC; Goodall et al., 1995). Briefly, leaves (0.5 g) were dissolved in 10 mL 80% ethanol, vortexed thoroughly and incubated at 70°C for 30 min. The mixture was centrifuged at 12,000 *g* for 10 min, and the supernatant was collected into a new tube. The centrifugation step was repeated twice to remove any debris. The supernatant was dried at 70°C and redissolved into 5 mL distilled water, and 1 mL was filtered through a membrane (0.22 μm) and transferred to a glass tube for HPLC analysis.

### Statistical Analysis

Three biological replicates were performed and the data were presented as the mean ± SE. Data difference analysis was carried out based on Student’s *t*-test (two-tailed analysis) at *P* < 0.01 or *P* < 0.05.

## Results

### *IbSWEET10* Encodes a Clade III SWEET Protein

**FIGURE 1:**
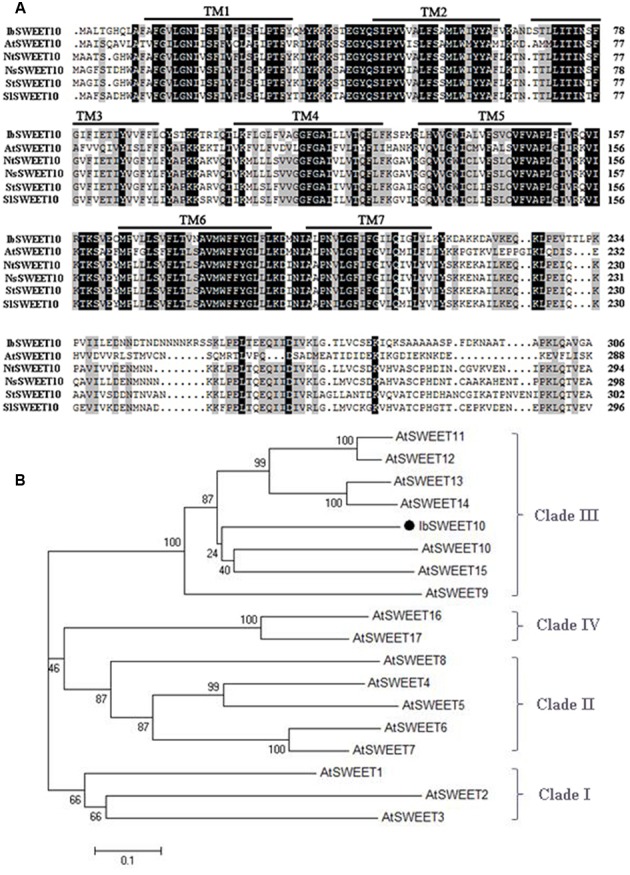
**Sequence analysis of *IbSWEET10*. (A)** Multiple sequence alignment of sugar transporters from *Ipomoea batatas* (IbSWEET10), *Arabidopsis thaliana* (AtSWEET10, Q9LUE3.1), *Nicotiana tabacum* (NtSWEET10, XP_016465379), *Nicotiana sylvestris* (NsSWEET10, XP_009762335), *Solanum tuberosum* (StSWEET10, XP_015164326) and *Solanum lycopersicum* (SlSWEET10, XP_004235333). Identical amino acids are denoted by dark shading, and conserved amino acids are indicated by a gray background. The seven transmembrane (TM) domains are outlined. **(B)** Phylogenetic analysis of SWEET proteins from *Ipomoea batatas* (IbSWEET10) and *Arabidopsis thaliana*.

A *SWEET* gene was cloned using the RACE method and we named it as *IbSWEET10* based on its closest phylogenetic relationship with *AtSWEET10* from *A. thaliana* (Eom et al., 2015). The cloned 1250-bp full-length *IbSWEET10* cDNA contains a 921-bp ORF that encodes a 306-amino acid polypeptide with a molecular weight of 34.1 kDa and a *p*I of 9.34. A search of the IbSWEET10 protein sequence in NCBI revealed two conserved MtN3 domains. The genomic sequence of *IbSWEET10* is 3269 bp long and contains six exons and five introns. The results of multiple sequence alignment show that the IbSWEET10 protein is a typical 7-TM SWEET (**Figure 1A**). Based on phylogenetic analysis, *IbSWEET10* has the closest relationship with *AtSWEET10* (**Figure 1B**), both belong to clade III of the SWEET family (Chen et al., 2010).

### *IbSWEET10* is Highly Expressed in Leaves

**FIGURE 2:**
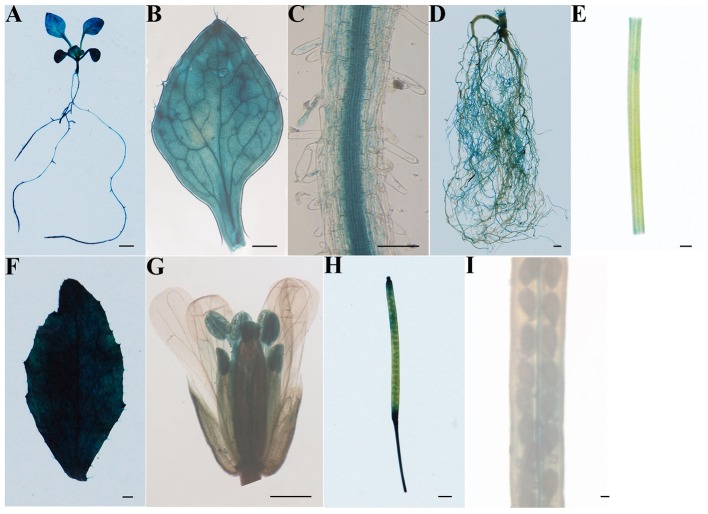
**Tissue-specific localization of the IbSWEET10 protein in transgenic *Arabidopsis* identified by histochemical analysis of β-Glucuronidase (GUS) activity driven by the *IbSWEET10* promoter. (A)** 2-week-old seedling. **(B,C)** Leaf and root of a 2-week-old seedling. **(D)** Mature root. **(E)** Stem. **(F)** Leaf. **(G)** Flower. **(H)** Silique. **(I)** Seed. Bars = 1 mm **(A,D–H)**, 0.05 mm **(C)**, 0.1 mm **(B,I)**.

Quantitative real-time PCR analysis revealed expression level of *IbSWEET10* was highest in leaves of 4-week-old *in vitro*-grown ND98 plants (Supplementary Figure S1A). High expression was also observed in leaves of 3-month-old ND98 plants from the field (Supplementary Figure S1B). As previously reported in sweet potato, the spatial expression pattern of a gene was correlated with its promoter activity in a heterologous system such as *Arabidopsis* (Noh et al., 2010, 2012; Tanabe et al., 2015). Therefore, the activity of the 1842 bp promoter including 5′UTR of *IbSWEET10* was investigated in different tissues of *Arabidopsis* using *gusA* gene as a reporter gene. In 2-week-old seedlings of *Arabidopsis*, GUS activity driven by P*_IbSWEET10_* was found in all of the tissues (**Figure 2A**), especially in the vascular tissues of leaves and roots (**Figures 2B,C**). In soil-grown mature *Arabidopsis* plants, the leaves showed the high GUS activity, but the reduced GUS activity was detected in roots, stems, flowers, siliques, and seeds (**Figures 2D–I**), which are consistent with *IbSWEET10* expression in sweet potato.

### IbSWEET10 is Localized to the Plasma Membrane

**FIGURE 3:**
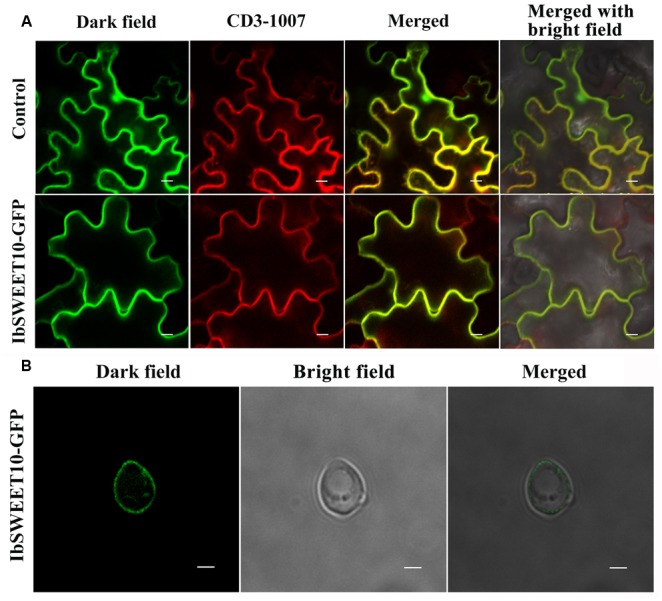
**Subcellular localization of the IbSWEET10 protein. (A)** The vector as a control (Upper row) and IbSWEET10-GFP (Lower row) protein were transiently expressed in *N. benthamiana* leaf hypodermal cells. The CD3-1007 marker was co-transformed to label the plasma membrane. Bars = 10 μm. **(B)** IbSWEET10-GFP protein was expressed in yeast cells. Bars = 2 μm.

In *Arabidopsis*, different SWEET proteins are localized to different cellular sites, and to date, four of the seven clade III AtSWEETs have been functionally shown to encode plasma membrane proteins (Seo et al., 2011; Chen et al., 2012; Lin et al., 2014; Chen L.Q. et al., 2015). Because IbSWEET10 is phylogenetically a clade III SWEET, we investigated whether it is also located in the plasma membrane by generating 2 × 35S_pro_-*IbSWEET10*-*GFP* construct and transient expression in *N. benthamiana* epidermal cells. As shown in **Figure 3A**, the green fluorescence emitted by the IbSWEET10-GFP fusion protein is coincident with the red fluorescence of the plasma membrane-localized marker CD3-1007, indicating that IbSWEET10 was localized to the plasma membrane. The subcellular localization in yeast further revealed that IbSWEET10 is a plasma membrane protein (**Figure 3B**).

### IbSWEET10 Transports Sucrose in Yeast

**FIGURE 4:**
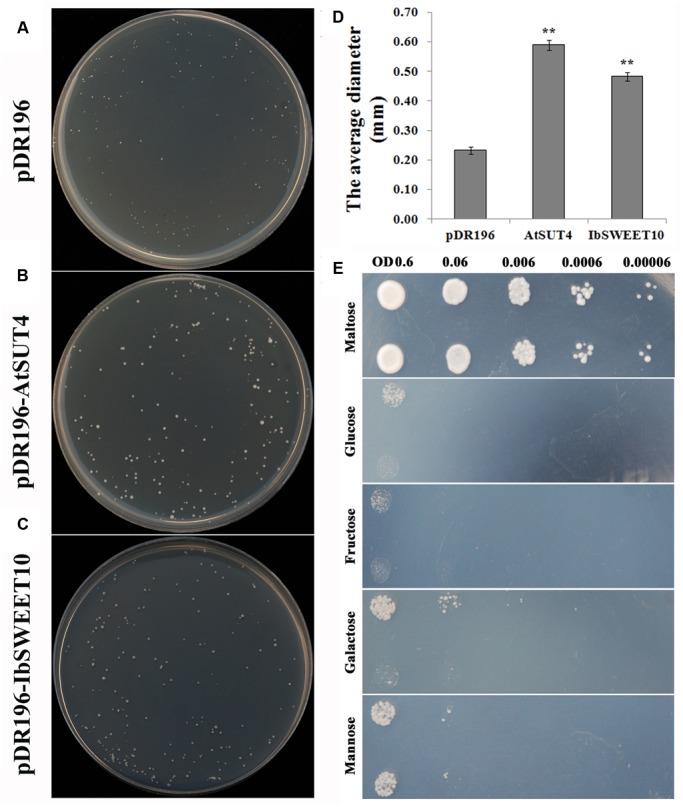
**Functional analysis of IbSWEET10 in the yeast mutant strain SUSY7/ura3 using sucrose as the main carbon source (A–D).** Yeast mutant strain EBY.VW4000 growth on media with different carbon sources **(E)**. **(A)** SUSY7/ura3 transformed with the empty pDR196 vector. **(B)** SUSY7/ura3 transformed with pDR196-*AtSUT4* as the positive control. **(C)** SUSY7/ura3 transformed with pDR196-*IbSWEET10*. **(D)** The average diameters of yeast colonies grown after 1 week. **(E)** Growth complementation test of EBY.VW4000 transformed with the 614 empty pDR196 vector (Upper row) and pDR196-*IbSWEET10* (Lower row) using different sugars as 615 carbon sources at degraded yeast OD value. Data are presented as the mean ± SE (*n* = 3). ^∗∗^ indicates a significant difference versus WT at *P* < 0.01 based on Student’s *t*-test.

To investigate whether *IbSWEET10* encodes a sugar transporter, the gene was expressed in the yeast mutant SUSY7/ura3, which is deficient in sucrose transport (Riesmeier et al., 1992). As shown in **Figures 4A–C**, SUSY7/ura3 cells expressing either *AtSUT4* or *IbSWEET10* grew better on sucrose media than the cells transformed with the empty pDR196 vector. The colonies transformed with *IbSWEET10* were 2.09-folds of ones transformed with the empty pDR196 vector in size (**Figure 4D**). This was a strong indication that *IbSWEET10* encodes a sucrose transporter. To examine whether IbSWEET10 transports hexose (glucose, fructose, galactose, and mannose), the yeast mutant EBY.VW4000 (Wieczorke et al., 1999), which is impaired in hexose transport, was transformed with *IbSWEET10*. However, no obvious signs of growth on media containing different hexoses as the main carbon source were observed (**Figure 4E**). These results revealed that *IbSWEET10* is a sucrose transporter rather than a hexose transporter.

### *IbSWEET10* Affects Sucrose Tolerance of Sweet Potato Plants

**FIGURE 5:**
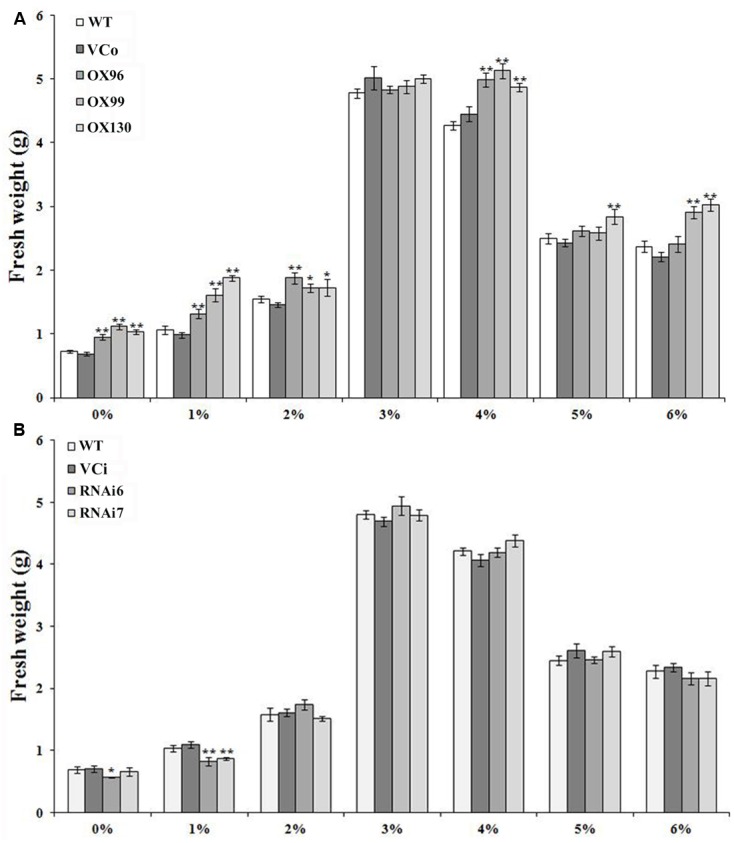
**Responses of transgenic Lizixiang plants and control plants cultured on MS media supplemented with different sucrose concentrations.** Fresh weights (FWs) were measured after 4 weeks of growth. **(A)** FWs of OX lines. **(B)** FWs of RNAi lines. Data are presented as the mean ± SE (*n* = 3). ^∗^ and ^∗∗^ indicate a significant difference versus WT at *P* < 0.05 or *P* < 0.01, respectively, based on Student’s *t*-test.

Transgenic Lizixiang lines were generated as described by Wang et al. (2016) (Supplementary Figure S2). At last, the 11 OX, 8 RNAi, 5 VCo and 5 VCi transgenic lines were obtained in this study. qRT-PCR analysis showed significant increases in the transcript level of *IbSWEET10* in three OX lines (OX96, OX99, and OX130; Supplementary Figure S3A) and dramatic decreases in two RNAi lines (RNAi6 and RNAi7; Supplementary Figure S3B) compared with WT and VCo/VCi. Under *in vitro* conditions, the sucrose concentration of the growth medium has an impact on the growth of plantlets (Jo et al., 2009). Because IbSWEET10 functions as a sucrose transporter, altered *IbSWEET10* expression could affect sucrose transport and thereby alter plant growth. To examine this possibility, *IbSWEET10* transgenic lines were grown on media containing different sucrose concentrations. Under a normal sucrose concentration (3%), both the OX and RNAi lines showed no difference in FW from WT and VCo/VCi. However, the OX lines exhibited enhanced tolerance to low (0–2%) or high (4%) sucrose concentrations compared with WT and VCo (**Figure 5A**). In contrast, low sucrose concentrations resulted in decreased FW in RNAi lines, whereas high sucrose concentrations caused little change compared with WT and VCi (**Figure 5B**).

### *IbSWEET10* Plays a Positive Role in Resistance to *F. oxysporum*

**FIGURE 6:**
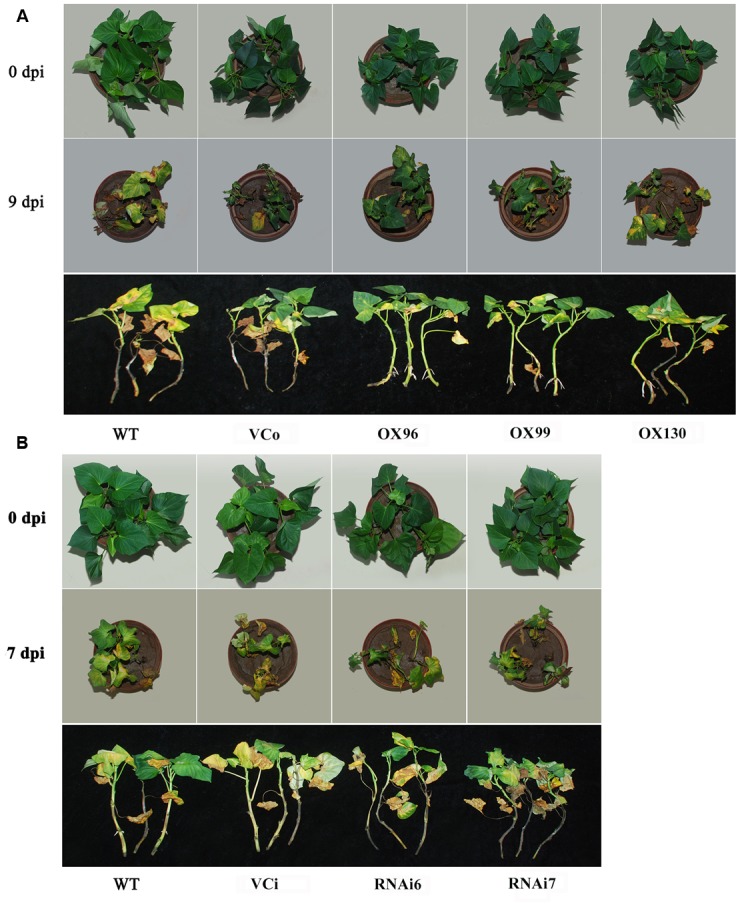
**Growth conditions of OX lines (A)** and RNAi lines of Lizixiang **(B)** versus control plants at 0 and 9 dpi of *F. oxysporum* infection.

**FIGURE 7:**
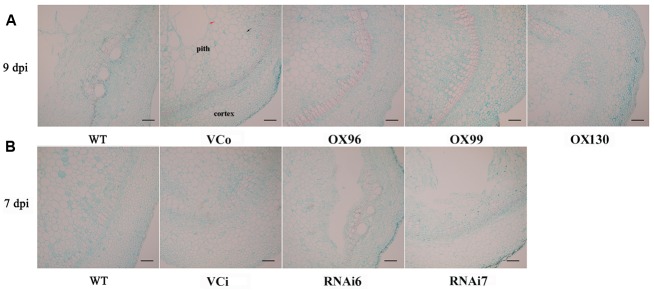
**Histological examination of transverse sections of stems of OX Lizixiang lines infected with *F. oxysporum* at 9 dpi (A)** and the RNAi lines at 7 dpi **(B)**. Black arrow indicated compact cells and red arrow indicated loose cells.

To verify whether *IbSWEET10* contributes to pathogen resistance, we first examined the changes in *IbSWEET10* expression in ND98 plants infected by *F. oxysporum*. qRT-PCR analysis indicated that *IbSWEET10* expression was significantly induced by the pathogen. The highest expression level was detected at 7 dpi: 3.46-fold of that at 0 dpi (Supplementary Figure S4). We further examined the severity of fusarium wilt in OX or RNAi lines at 9 or 7 dpi, respectively. At 9 dpi, almost half of the WT and VCo leaves were withered and yellow, whereas only a few OX leaves showed this symptom. The length of stem browning of the OX lines was also shorter than that of WT and VCo. Some new roots were formed on the OX stems, but no root formation was observed in WT and VCo (**Figure 6A**). Furthermore, RNAi lines exhibited more severe symptoms compared with WT and VCi, most of the leaves became wilted and yellow, the stems turned brown to black, and no root formation was found on the stems (**Figure 6B**). Histological observation of stems showed that the cells in the pith of WT and VCo were deformed and became loosely arranged, whereas the stem structure of OX lines remained intact, with compact cells in the pith and cortex (**Figure 7A**). Cross-sectional observation of the stems of RNAi lines exhibited a destroyed pith structure and distortion of cortex cells, which were not serious in WT and VCi (**Figure 7B**). These results indicate that *IbSWEET10* plays a positive role in enhancing sweet potato resistance to *F. oxysporum*.

### *IbSWEET10* Is Associated with Sugar Contents in Sweet Potato

**FIGURE 8:**
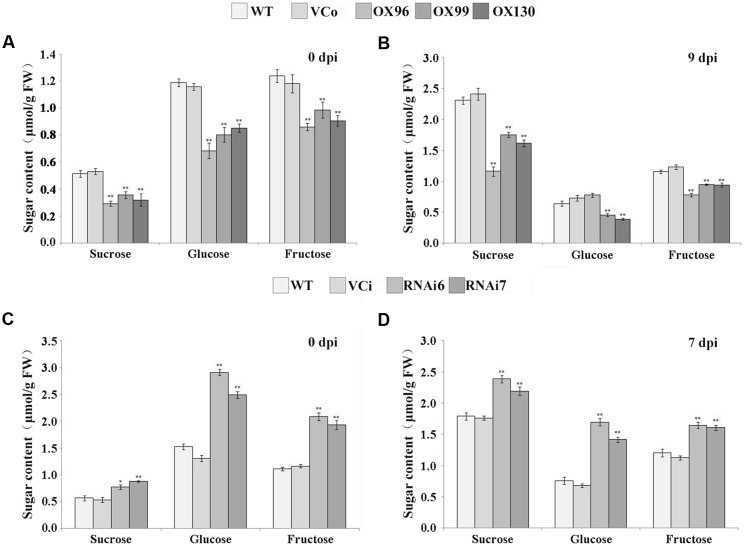
**Sugar content in leaves of OX lines (A,B)** and RNAi lines **(C,D)** of Lizixiang at 0 and 9 dpi of *F. oxysporum* infection, respectively. Data are presented as the mean ± SE (*n* = 3). ^∗^ and ^∗∗^ indicate a significant difference versus WT at *P* < 0.05 or *P* < 0.01, respectively, based on Student’s *t*-test.

The carbohydrate status of plants affects the development/resistance of disease and vice versa (Berger et al., 2007; Gamm et al., 2011; Li et al., 2014). Because we identified the sucrose transporter activity of IbSWEET10, the altered resistance of transgenic plants to *F. oxysporum* was most likely caused by an uneven distribution of sucrose and other soluble sugars. Therefore, the sucrose, glucose and fructose contents in the leaves of transgenic and control plants were examined. For OX lines, a significant decrease in each of these three sugars was observed at both 0 and 9 dpi compared with WT and VCo (**Figures 8A,B**). However, RNAi lines contained higher levels of each sugar at 0 and 7 dpi compared with WT and VCi (**Figures 8C,D**). We also noticed that compared with 0 dpi, the sucrose content in each plant line was increased at 9 or 7 dpi.

## Discussion

### The Plasma Membrane Protein IbSWEET10 Is a Sucrose Transporter

Sugars Will Eventually be Exported Transporters have been demonstrated to have sugar transport capacity, mainly transporting sucrose and glucose in many plants (Chen et al., 2010; Chen L.Q. et al., 2015). In the present study, assays using sucrose transport-deficient yeast SUSY7/ura3 cells revealed that IbSWEET10 is a sucrose transporter (**Figure 4**). Based on expression in hexose transport-deficient yeast EBY.VW4000 cells, IbSWEET10 did not transport glucose, fructose, galactose or mannose (**Figure 4D**). This result is consistent with other reported SWEETs that belong to clade III (Chen L.Q. et al., 2015).

It is reported that the physiological role of sucrose transporters can be predicted through analysis of their tissue-specific expression and subcellular localization. The most possible route of sucrose transport into the SE/CC might begin directly from its production site in mesophyll cells through the apoplasm and into phloem parenchyma cells (Chen et al., 2012). Analysis of the GUS expression patterns of P*_IbSWEET10_*-GUS-transgenic *Arabidopsis* revealed that *IbSWEET10* is more abundantly expressed in leaves of mature plants, especially in vascular tissues (**Figure 2F**). This finding indicates that IbSWEET10 functions to export sucrose from source leaves and participates in phloem loading. A two-step model of sucrose apoplasmic phloem loading shows that sucrose is exported by SWEETs residing in the plasma membrane of phloem parenchyma cells and feeds the sucrose transporter SUT in the SE/CC (Chen et al., 2012). Our study reveals that IbSWEET10 is located in the plasma membrane (**Figure 3**). Thus, we suggest that IbSWEET10 participates in the first step of sucrose apoplasmic phloem loading across the plasma membrane.

### Overexpression of *IbSWEET10* Increases Tolerance of Sweet Potato to Sucrose

Plants growing under tissue culture conditions are semiautotrophic, and supplementation of sucrose in the medium meets the energy demand for growth and physiological functions (Hazarika, 2003). The *atsweet11;12* mutants show reduced root length when germinated on sugar-free media (Chen et al., 2012) and *atsuc2* mutant seedlings are smaller than wild-type under the same conditions (Gottwald et al., 2000). In this study, a significant increase in fresh weigh of the OX plants was observed compared with the controls under low sucrose concentration (0–2%), suggesting that overexpression of *IbSWEET10* increases the efflux of sucrose from leaves and promotes the plant growth (**Figure 5A**). Under high sucrose concentration (4%), the OX plants also exhibited the increased FW compared with the controls, thinking that its overexpression might facilitate sucrose transport into the OX plant roots from leaves and medium, which induces osmotic stress balance in the rhizosphere (Jo et al., 2009; Chen L.Q. et al., 2015). However, when expression of *IbSWEET10* was suppressed, low sucrose concentrations in the medium caused the decreased FW in RNAi plants. No significant difference in FW was observed between RNAi plants and controls at high sucrose concentrations (**Figure 5B**). One possible explanation for this result is that reduced expression of *IbSWEET10* might lead to impaired export of sucrose from leaves and thus negatively affect the plant growth (Burkle et al., 1998). When the sucrose supply was sufficient, the RNAi plants were able to acquire sucrose from the medium for growth.

### Overexpression of *IbSWEET10* Enhances Resistance of Sweet Potato to *F. oxysporum*

Access to nutrients from hosts for reproduction is the primary objective of infection (Chen et al., 2010), and sugars are among the most important nutrients providing energy for pathogen growth. For many diseases caused by soil pathogens, the nutrients provided by the host are considered to be a main factor in deciding the success or failure of symptomatic development (Hancock and Huisman, 1981). The observed high incidence of *Fusarium* with the *shrunken-2* mutation was proposed to result from the elevated sugar content in kernels (Headrick et al., 1990). Additionally, *AtSWEET2* activity reduced sugar availability in the rhizosphere, thus contributing to *Pythium* resistance (Chen H.Y. et al., 2015). Thus, lack of carbohydrates will limit the growth of a pathogenic fungus and reduce the ability of the pathogen to infect plants.

Alteration of the expression levels of sucrose transporter genes might cause changes in the sugar content in plants. Leaves of *atsweet11:12* double mutants accumulate high levels of sugars, indicating an impaired ability to export sugar from the leaves (Chen et al., 2012). In addition, disruption of *AtSUC4* or *AtSUC2* caused sucrose accumulation in source leaves possibly by interfering with phloem loading (Gong et al., 2015), and the sucrose concentration in the vacuole of *PatSUT4*-RNAi lines was higher than in WT under well-watered conditions (Frost et al., 2012). In our study, overexpression of *IbSWEET10* might enhance sugar export from leaves, which can explain the lower sugar contents in the leaves of OX plants. The opposite was observed for RNAi plants (**Figure 8A**). Long-distance transport of sucrose between leaves and roots occurs in the phloem sieve elements of the stem vascular system (Williams et al., 2000), and blocked phloem loading causes more accumulation of sugars in leaves (Chen et al., 2012). After *F. oxysporum* infection, the stems of OX plants displayed only slight damage and phloem loading block, which facilitated the transport of sugars from the leaves compared with WT, VCo, VCi and RNAi plants which showed serious damage and phloem loading block (**Figure 7**). As a result, the sucrose content was significantly decreased in the OX leaves than in others after *F. oxysporum* infection (**Figures 8B,D**). These results indicated that *IbSWEET10* plays an important role in sugar transport in sweet potato plants.

The soil-borne *F. oxysporum* proliferates in the vascular of sweet potato stems and then spreads to the leaves, which causes wilting of stems and leaves. Therefore, the lower level of sugars in the OX stems and leaves might limit the proliferation of the pathogen. Meanwhile, closely arranged cell structure of OX stems might prevent the invasion and spread of *F. oxysporum*. As a result, the OX plants exhibited the enhanced resistance to *F. oxysporum* (**Figure 6**).

In addition, the overexpression of *OsSWEET11*, -*13*, and -*14* enhanced the pathogenicity of *X. oryzae* in rice (Yang et al., 2006; Antony et al., 2010; Liu et al., 2011). The *AtSWEET2-*overexpressing *Arabidopsis* plants showed the increased resistance to *P. irregulare* (Chen H.Y. et al., 2015). Our results revealed that overexpression of *IbSWEET10* enhanced the resistance of sweet potato to *F. oxysporum*. Therefore, it is thought that different members of the SWEET gene family differentially function in the responses to plant pathogens.

## Conclusion

A member of the SWEET gene family named *IbSWEET10*, a sucrose transporter gene, was cloned from the sweet potato line ND98. Its overexpression significantly enhanced resistance to *F. oxysporum* in transgenic sweet potato plants. We suggest that the reduced sugar levels caused by overexpression of *IbSWEET10* may decrease the carbohydrate supply for the pathogen and thus contribute to enhanced resistance to *F. oxysporum* in sweet potato. The *IbSWEET10* gene has the great potential to be used for improving the resistance to *F. oxysporum* in sweet potato and other plants.

## Author Contributions

SH, QL, and YL conceived and designed the experiments. YL, YW, HuZ, and QZ performed the experiments. YL, SH, YW, and HoZ analyzed the data and wrote the paper. SH and QL revised the paper. All authors read and approved the final version of the paper.

## Conflict of Interest Statement

The authors declare that the research was conducted in the absence of any commercial or financial relationships that could be construed as a potential conflict of interest.

## Footnotes

1 http://www.ncbi.nlm.nih.gov/projects/gorf/
