# Supplementary material for: The Plasma Membrane-Localized Sucrose Transporter IbSWEET10 Contributes to the Resistance of Sweet Potato to Fusarium oxysporum
Source: Front Plant Sci. 2017 Feb 14;8:197. doi: 10.3389/fpls.2017.00197 (PMC5306249; doi:10.3389/fpls.2017.00197)
Supplement: Supplementary file 1 [file Data_Sheet_1.docx]

Supplementary Material

**The plasma membrane-localized sucrose transporter** **IbSWEET10 contributes to the resistance of sweet potato to *Fusarium oxysporum***

Yan Li^1, †^, Yannan Wang^1, †,^ Huan Zhang^1^, Qian Zhang^1^, Hong Zhai^1^, Qingchang Liu^1,*^ & Shaozhen He^1,*^

^1^Beijing Key Laboratory of Crop Genetic Improvement/Laboratory of Crop Heterosis and Utilization, Ministry of Education, China Agricultural University, Beijing 100193, China

^†^ These authors contributed equally to this work.

^*^Correspondence and requests for materials should be addressed to S. H. (email: [sunnynba@cau.edu.cn](mailto:sunnynba@cau.edu.cn)) and Q. L. (email: liuqc@cau.edu.cn)

Supplementary Table S1. Sequences of the primers used in this study

| **Primer name** | **Primer sequence (5’-3’)** |
| --- | --- |
| 5GSP1 | GTAGAATGTTGGCAGTGGAGAAAG |
| 5GSP2 | AAAGCAAAAGCCAATTGATGACC |
| 3GSP1 | ATGGCTCTCACTGGTCATCAATT |
| 3GSP2 | TCATCTCATTCATCGTCTTCCTTTC |
| GW1 | ATGAGAGTAAATATAAGGAAAGGAAAGCAGCA |
| GW2 | AAAGCCAATTGATGACCAGTGAGAGCC |
| QSWEET10-F ^a^ | TTTCTGTTCAAAAGTCCGATGC |
| QSWEET10-R | CATTCCACGCTCTTGGTGC |
| Qactin-F | AGCAGCATGAAGATTAAGGTTGTAGCAC |
| Qactin-R | TGGAAAATTAGAAGCACTTCCTGTGAAC |
| 196-F-*Sma*I (*IbSWEET10*) | TCCCCCGGGATGGCTCTCACTGGTCATCAA |
| 196-R-*Xho*I (*IbSWEET10*) | CCGCTCGAGTTAAGCTCCCACAGCCTGAA |
| 196-F-*Sma*I (*AtSUT4*) | TCCCCCGGGATGGCTACTTCCGATCAAGATCGC |
| 196-R-*Xho*I (*AtSUT4*) | CCGCTCGAGTCATGGGAGAGGGATGGGCTT |
| 83S-F-*Spe*I | GACTAGTATGGCTCTCACTGGTCATCAA |
| 83S-R-*Asc*I | AGGCGCGCCAAGCTCCCACAGCCTGAAG |
| SG-F-*SmaI* | TCCCCCGGGATGGCTCTCACTGGTCATCAA |
| SG- R-*XhoI* | CCGCTCGAGTTAGTGGTGGTGGTGGTGG |
| 162-F-*Pac*I | CCTTAATTAAATTTTTATACAATATGAGTAGTCCCC |
| 162-R-*Asc*I | AGGCGCGCCAGTGTTGTTCTCTTAGTTAACTGTAAGAA |
| OS-F-*Xba*I | GCTCTAGAATGGCTCTCACTGGTCATCAA |
| OS-R-*Sac*I | CGAGCTC TTAAGCTCCCACAGCCTGAA |
| Si-UF-*Xho*I | CCGCTCGAGAGCTTCCTGAACTCACCGAG |
| Si-UR-*Swa*I | GCATTTAAATCTCCCACAGCCTGAAGCTTA |
| Si-DF-*BamH*I | CGGGATCCCTCCCACAGCCTGAAGCTTA |
| Si-DR-*Xba*I | GCTCTAGAAGCTTCCTGAACTCACCGAG |
| T35-F | TTGATGTGATATCTCCACTGACG |
| TS-R | GGTAGAGACCGATCTGGAGGA |
| int-F | CAACCACAAAAGTATCTATGAGCCT |
| int-R | TTCACATGTCAGAAACATTCTGATG |

^a^ Primers used for the qRT-PCR reactions begin with the letter “Q”.


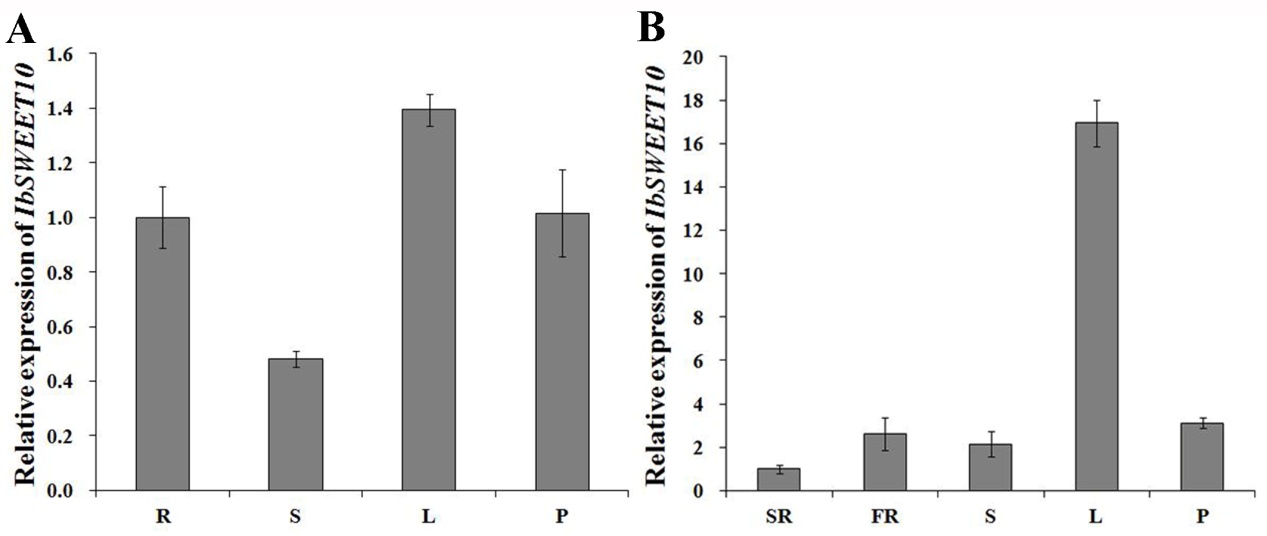


Supplementary figure S1. Expression analysis of *IbSWEET10* in sweetpotato line ND98 by real-time quantitative PCR. (A) Transcript levels of *IbSWEET10* in roots (R), stems (S), leaves (L) and petioles (P) of 4-week-old in vitro-grown ND98 plants. (B) Transcript levels of *IbSWEET10* in different tissues of 3-month-old ND98 plants in the field. SR, storage roots; FR, fibrous roots; S, stems; L, leaves; P, petioles. Data are presented as the mean + SE (n=3).


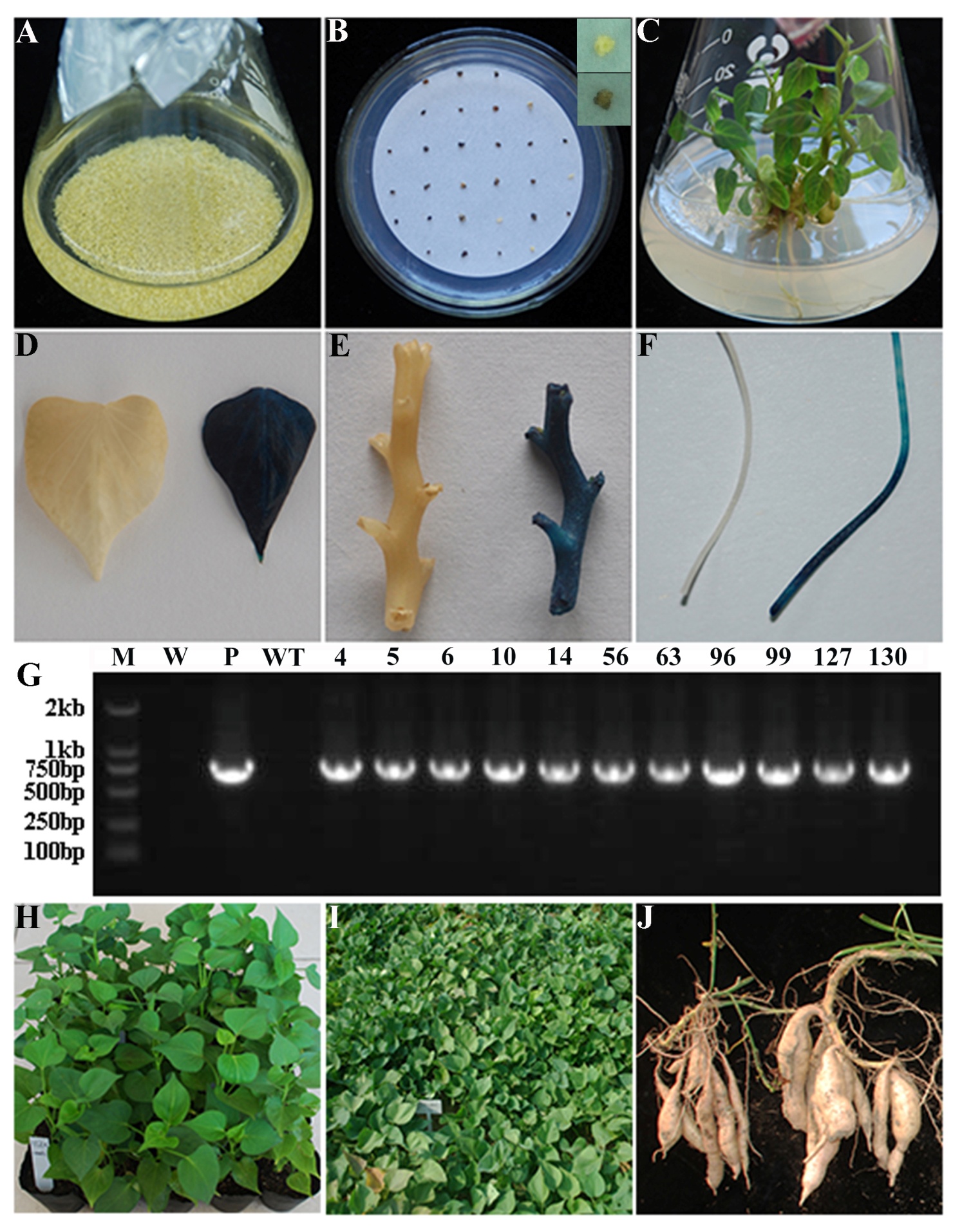


Supplementary figure S2. Production of transgenic sweet potato plants overexpressing the *IbSWEET10* gene. (A) Embryogenic suspension cultures of Lizixiang rapidly proliferating in MS medium containing 2.0 mg L^-1^ 2,4-D. (B) Phosphinothricin (PPT)-resistant calli (bright yellow) formed on MS medium with 2.0 mg L^-1^ 2,4-D, 300 mg L^-1^ cefotaxime sodium and 0.5 mg L-1 PPT after 8 weeks of selection. The insert pictures showed the resistant and sensitive calli. (C) Regeneration of plantlets from PPT-resistant calli on MS medium with 1.0 mg L-1 ABA and 300 mg L^-1^ cefotaxime sodium. (D-F) GUS expression in leaves, stems and roots of a transgenic plants and no GUS expression in WT, respectively. (G) PCR analysis of GUS-positive plants. Lane M: BL2000 DNA marker; Lane W: water as the negative control; Lane P: plasmid pCAMBIA3301-*IbSWEET10* as the positive control; Lane WT: WT as the negative control; Lanes L4-130: GUS-positive plants. (H) Transgenic plants grown in the greenhouse. (I) Transgenic plants grown in the field. (J) Storage roots of transgenic sweet potatoes.


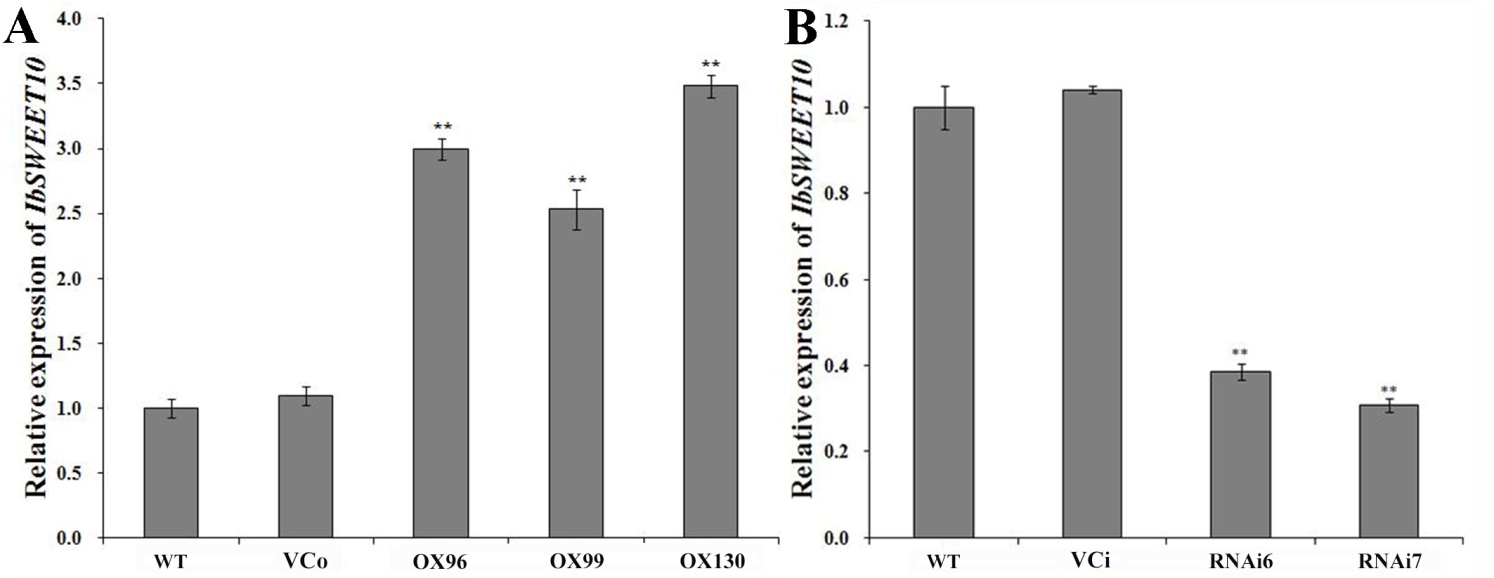


Supplementary figure S3. Transcript levels of *IbSWEET10* in OX lines (A) and RNAi lines (B). The results are expressed as relative values with respect to Lizixiang (WT), which was set to 1.0. Data are presented as the mean + SE (n=3). ** indicates a significant difference versus WT at *P* < 0.01 based on Student’s *t*-test.


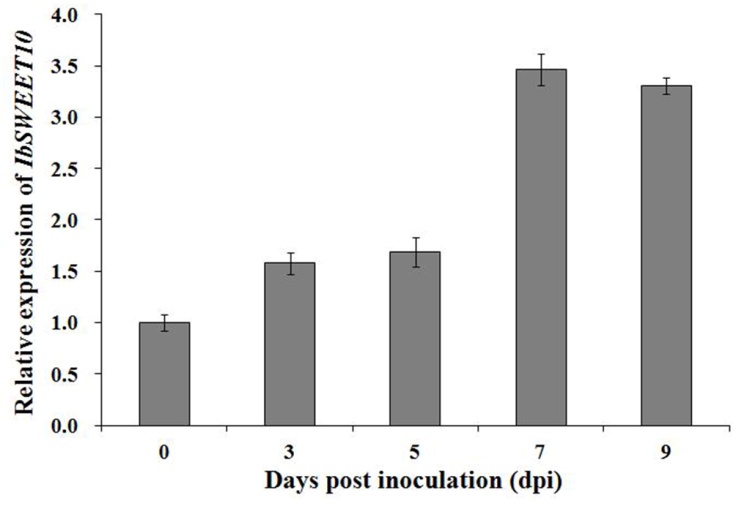


Supplementary figure S4. Transcript levels of *IbSWEET10* in ND98 plants at different dpi of *F. oxysporum* infection. Cuttings of 20 cm in length were soaked in spore suspension for 30 min and grown in sterile sandy loam. Leaves were sampled at 0, 3, 5, 7 and 9 dpi to analyse expression of *IbSWEET10*. Data are presented as the mean + SE (n=3).
